# Correction: Antibiofilm activity of *Parkia javanica* against *Pseudomonas aeruginosa*: a study with fruit extract

**DOI:** 10.1039/d5ra90048d

**Published:** 2025-05-01

**Authors:** Antu Das, Manash C. Das, Padmani Sandhu, Niranjan Das, Prosun Tribedi, Utpal C. De, Yusuf Akhter, Surajit Bhattacharjee

**Affiliations:** a Department of Molecular Biology & Bioinformatics, Tripura University Suryamaninagar Tripura 799022 India sbhattacharjee@gmail.com; b Centre for Computational Biology and Bioinformatics, School of Life Sciences, Central University of Himachal Pradesh Shahpur, District Kangra Himachal Pradesh 176206 India; c Department of Chemistry, Netaji Subhash Mahavidyalaya Udaipur Tripura 799114 India; d Department of Microbiology, Don Bosco University Guwahati Assam–781017 India; e Department of Chemistry, Tripura University Suryamaninagar Tripura 799022 India

## Abstract

Correction for ‘Antibiofilm activity of *Parkia javanica* against *Pseudomonas aeruginosa*: a study with fruit extract’ by Antu Das *et al.*, *RSC Adv.*, 2017, **7**, 5497–5513, https://doi.org/10.1039/C6RA24603F.

The authors regret an inadvertent error in Fig. 5B, where an overlap was unintentionally introduced between Fig. 4C(iv) and Fig. 5B(v) during the figure preparation process.

The corrected Fig. 5B and associated caption is shown here.

**Fig. 5:**
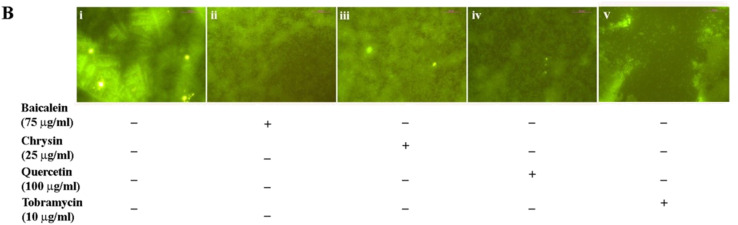
Attenuation in biofilm formation was further observed using fluorescence microscopy and the best representative images of 5 different individual sets of experiments are presented [B(i–v)].

An independent expert has viewed the corrected image and has concluded that they are consistent with the discussions and conclusions presented.

The Royal Society of Chemistry apologises for these errors and any consequent inconvenience to authors and readers.

